# Prognostic significance of systemic immune inflammation index in patients with urothelial carcinoma: a systematic review and meta-analysis

**DOI:** 10.3389/fonc.2024.1469444

**Published:** 2024-12-23

**Authors:** Lei Zheng, Zuoping Wang, Yunxiang Li, Si Ge, Zhiqiang Zeng, Lijian Gan, Chunyang Meng, Kangsen Li

**Affiliations:** ^1^ Department of Urology, Nanchong Central Hospital, The Second Clinical College, North Sichuan Medical College (University), Nanchong, Sichuan, China; ^2^ Department of Urology, School of Clinical Medicine, Southwest Medical University, Luzhou, Sichuan, China

**Keywords:** meta-analysis, urothelial carcinoma, prognosis, systemic immune inflammation index, SII

## Abstract

**Objective:**

This review assessed the prognostic significance of the systemic immune inflammation index (SII) in patients with urothelial carcinoma.

**Methods:**

We performed a systematic review and cumulative meta-analysis of the primary outcomes according to the PRISMA criteria, and assessed study quality. Seven databases were searched: Embase, PubMed, Cochrane Library, Web of Science, China National Knowledge Infrastructure, Wanfang, and SinoMed, from the creation of each database until October 2024.

**Results:**

The meta-analysis included 31 studies, including 14,437 patients with urothelial carcinoma. A low SII was significantly associated with better recurrence-free survival (RFS) (HR = 1.37, 95%CI (1.19, 1.56), P < 0.05), cancer-specific survival (CSS) (HR = 1.87, 95%CI (1.50, 2.34), P < 0.05), and overall survival (OS) (HR = 1.42, 95%CI (1.23, 1.64), P < 0.05). In addition, subgroup analysis found that higher SII was associated with poorer prognosis regardless of treatment regimen, tumor type, or SII cutoff, and that high SII was an important prognostic biomarker in the UC population.

**Conclusion:**

A low SII may be associated with better RFS, CSS, and OS. The SII can be used as a is a potentially noninvasive and promising prognostic indicator for urothelial carcinoma; however, further studies with appropriate designs and larger sample sizes are needed to verify these findings.

## 1 Introduction

Urothelial carcinoma (UC) is one of the most prevalent malignant neoplasms of the genitourinary system and can arise in any segment of the transitional epithelium of the urinary tract, including the renal pelvis, ureter, bladder, and urethra. This cancer can be categorized into Upper Tract Urothelial Carcinoma (UTUC) and Bladder Urothelial Carcinoma (BC), based on its location. The global incidence and mortality rates for UC have been consistently rising annually (1). Take bladder cancer as an example, about 614,000 new cases and about 220,000 deaths were reported globally in 2022, of which about 90% were bladder urothelial carcinoma, making it one of the most lethal cancers in the world (2–4). With the aging of the global population, the incidence and mortality rates of UC are projected to continue to rise, and the prognosis for patients with UC remains generally unfavorable. Consequently, there is an urgent need to identify effective biomarkers that can enhance the diagnostic accuracy, therapeutic evaluation, and prognostic assessment of UC in clinical settings.

An increasing body of research has underscored the intricate relationship between inflammation and the initiation, progression, and spread of tumors, with chronic inflammation being implicated as a risk factor for tumorigenesis (5, 6). Currently, several inflammatory biomarkers are being considered as potential diagnostic and prognostic tools for cancer, including lymphocyte, neutrophil, and platelet counts, as well as C-reactive protein (CRP) and the neutrophil-lymphocyte ratio (NLR).While CRP and erythrocyte sedimentation rate (ESR) are accessible, cost-effective markers of systemic inflammation, their lack of specificity means they can be influenced by non-neoplastic conditions such as infections and autoimmune diseases (7, 8). The Systemic Immunoinflammatory Index (SII), a novel marker derived from neutrophil, lymphocyte, and platelet counts, has demonstrated its prognostic significance across a spectrum of cancers (9, 10). Numerous studies have indicated the potential utility of SII in predicting urothelial cancer outcomes, advocating for its incorporation into routine assessments for these patients. For instance, elevated SII levels have been shown to be an independent predictor of adverse prognosis and response to BCG therapy in patients with uroepithelial carcinoma (11). Furthermore, the role of immune checkpoint inhibitors (ICIs) in urothelial carcinoma treatment and their associated biomarkers for efficacy and prognosis have been a focus of recent research reviews (12). As an emerging biomarker, SII has demonstrated considerable promise in evaluating prognosis and monitoring treatment responses in urothelial carcinoma. Despite the exploration of SII’s prognostic significance in UC patients, findings have been inconsistent (13–15). Consequently, this meta-analysis seeks to evaluate the prognostic significance of SII in UC based on the extant evidence.

## 2 Methods

### 2.1 Search strategy

This study was registered in PROSPERO, and followed the PRISMA meta-analysis guidelines (16, 17) and AMSTAR guidelines (18) for quality assessment. Two researchers independently conducted systematic online literature retrieval and data extraction. Electronic science databases were searched, including PubMed, Embase, Cochrane Library, Web of Science, China National Knowledge Infrastructure (CNKI), Wanfang Database, and China Biomedical Database, from their inception to October 2024. The search terms were as follows: (“Systemic immune inflammation index” OR “SII”) AND (“Urothelial carcinoma” OR “Transitional Cell Carcinoma*”), and all searches were performed using a combination of MeSH words and free words. Additionally, relevant systematic reviews and references from the included studies were manually identified and retrieved for further analysis.

### 2.2 Eligibility criteria

The inclusion criteria were identified according to the PICOS (population, intervention, comparator, outcomes, and study) criteria. The inclusion criteria were formulated as shown below: (i)P (population): Patients whose UC was confirmed pathologically. (ii)I (intervention): The SII level was examined for UC patients, and studies identified a cutoff value of SII for stratifying patients as low/high SII. (iii)C (comparator): UC patients with high SII level. (iv) O (outcome): Studies report associations between SII and UC survival outcomes; During the defined follow-up period, patients had at least one of the following survival outcomes: cancer-specific survival (CSS), overall survival (OS), and relapse-free survival (RFS); and provided hazard ratios (HR) and corresponding 95% confidence intervals (CI)for survival outcomes or provided sufficient data to calculate them.(v) S (study design): Cohort studies, including prospective and retrospective cohorts published in English or Chinese.

The exclusion criteria were as follows: (i) studies on cell lines, tissues, or animals; (ii) studies without necessary data; (iii) duplicate articles; (iv) case series, review articles, letters, editorials, or reviews; and (v) studies involving patients without urothelial carcinoma.

### 2.3 Quality evaluation

Based on the results of the identification process, we used the Newcastle–Ottawa Scale (NOS) to assess the quality of the included studies (19). This scale includes three areas: selection, comparability, and exposure. It assigns a score ranging from zero to nine stars, with studies receiving six or more stars being classified as high quality.

### 2.4 Data extraction

Two researchers employed a standardized data extraction form to meticulously collect the following details from the eligible studies: authors, year, country, study design, sample size, treatment methods, median follow-up, survival outcome, cutoff value, and tumor location. When continuous variables were reported as median and range in the main literature, we calculated the mean and SD (20).

### 2.5 Data analysis

Data analysis was conducted using Stata version 16. The HR and 95% CI of the multivariate analysis in each study was used to assess the importance of the SII in the prognosis of UC patients. In a meta-analysis, when the effect index is the HR, the risk ratio is usually taken as the logarithm of the effect value. Therefore, we used Stata 16 to find the logarithmic values of HR and the upper and lower limits of the 95% CI, and then performed a meta-analysis. The other parameters were extracted directly from the original study without conversion. We performed the Q and χ^2^ tests to value the heterogeneity between the included studies. If I^2^ > 50%, the differences between the studies were considered significant and random effect models were used. Otherwise, a fixed effects model was selected. In addition, sensitivity analyses were performed. The optimal cutoff value of the systemic inflammatory immune index was determined based on the receiver operating characteristic curve. Subgroup analyses were performed on tumor location, treatment modality, and SII cutoff values.

## 3 Results

### 3.1 Research description

**Figure 1:**
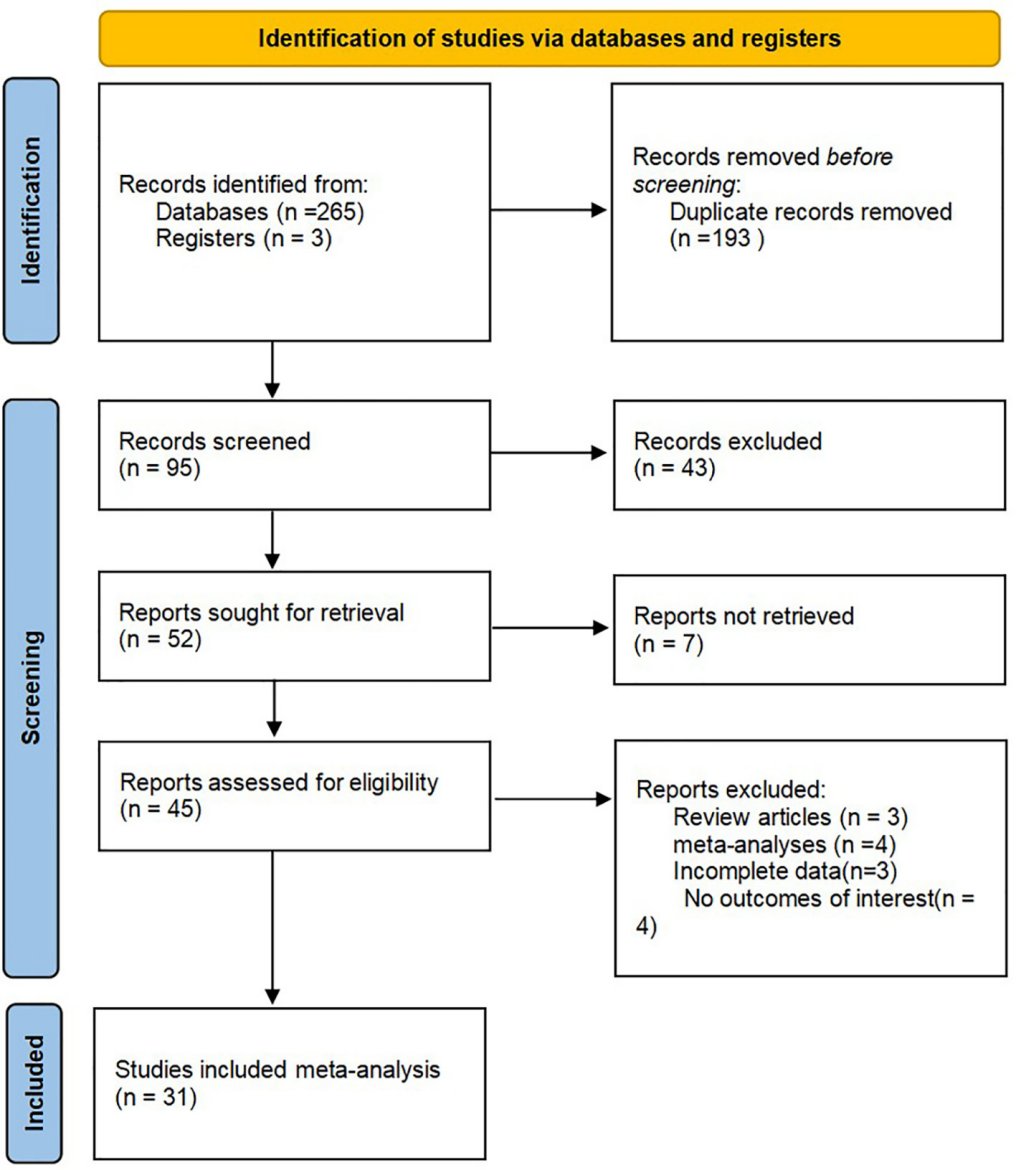
Flow diagram of the study selection process.

Through the search process, 268 studies were screened from the established databases, with an additional three studies discovered through manual searches. We utilized document management software to eliminate a total of 193 duplicate articles. After reviewing the titles and abstracts, 43 articles were excluded after reading titles and abstracts, seven were not retrieved, and 95 were included in careful reading, excluding four studies with no outcomes of interest, three systematic reviews, four meta-analyses, and three with incomplete data. Ultimately, 31 studies including 14,437 patients were included in the meta-analysis (21–51). A detailed systematic search process is presented in **Figure 1**. The baseline data of the included studies, including authors, year, country, study design, sample size, treatment methods, survival outcomes, cutoff values, and tumor locations are presented in **Table 1**.

**Table 1:** Baseline characteristics of the included studies.

Authors	Year	Country	Study design	Sample size	Treatment methods	Survival outcome	Cutoff value	Tumor type
Grossmann NC et al. (23)	2022	Multicenter	Retrospective	4335	surgery	RFS^c^ CSS^b^ OS^a^	610	UCB^d^
Kobayashi S et al. (40)	2021	NR	Retrospective	103	surgery+adjuvant therapy	CSS OS	520	UTUC^e^
Mori K et al. (28)	2021	Multicenter	Retrospective	2492	surgery+adjuvant therapy	RFS CSS OS	485	UTUC
Chien TM et al. (39)	2021	China	Retrospective	376	surgery	CSS	485	UTUC
Katayama S et al. (25)	2021	Multicenter	Retrospective	1117	surgery+adjuvant therapy	RFS CSS OS	580	NMIBC^f^
Zhang et al. (50)	2022	China	Retrospective	110	surgery+adjuvant therapy	RFS	410.3	UTUC
Huang et al. (51)	2022	China	Retrospective	119	surgery	RFS	370.5	NMIBC
Jan HC et al. (24)	2018	China	Retrospective	424	surgery	RFS CSS OS	580	UTUC
Zheng Y et al. (37)	2020	China	Retrospective	272	surgery	RFS CSS OS	672.44	UTUC
Xingxing Tang et al. (30)	2020	China	Retrospective	79	surgery	RFS OS	463.56	BC
Wentao Zhang et al. (49)	2019	China	Retrospective	70	surgery	OS	507	BC
Zhi-Bin Ke et al. (26)	2021	China	Retrospective	184	surgery+adjuvant therapy	RFS	NR	NMIBC
Peng Liu et al. (27)	2022	China	Retrospective	183	adjuvant therapy	RFS	514	NMIBC
Huifeng Bi et al. (38)	2020	China	Retrospective	387	surgery+adjuvant therapy	OS CSS	467.76	NMIBC
Li Deng-Xiong et al. (21)	2023	China	Retrospective	197	adjuvant therapy	RFS	557	NMIBC
G Fornarini et al. (44)	2021	China	Retrospective	267	adjuvant therapy	OS	1375	UC
Ali Yılmaz et al. (42)	2020	Italy	Retrospective	152	NR	OS	768	MIBC^g^
Shimpei Yamashita et al. (41)	2021	NR	Retrospective	237	surgery	CSS OS	438	BC
Shiyu Zhang et al. (34)	2022	Japan	Retrospective	725	surgery	OS RFS	554	BC
Li Ding et al. (22)	2023	China	Retrospective	416	surgery	RFS	505	NMIBC
Patrik Palacka et al. (47)	2021	China	Retrospective	181	adjuvant therapy	OS PFS	705	UC
Sacit Nuri Gorgel et al. (45)	2019	NR	Retrospective	191	surgery	CSS OS	843	MIBC
Chengbo Wang et al. (31)	2023	NR	Retrospective	222	adjuvant therapy	RFS	707	NMIBC
Hasan Yilmaz et al. (33)	2022	China	Retrospective	241	surgery	OS RFS	1228	BC
Ruining Zhao et al. (36)	2021	Türkiye	Retrospective	216	surgery	RFS	276.85	BC
Abolfazl Salari et al. (48)	2024	NR	Retrospective	187	surgery	OS	410	MIBC
Zhenkai Luo et al. (46)	2023	China	Retrospective	99	surgery	OS	470	UTUC
Pierluigi Russo et al. (29)	2023	NR	Retrospective	193	surgery	RFS CSS OS	640	BC
Xiaoping Zhang et al. (35)	2023	China	Retrospective	94	surgery	OS	863	BC
Michele Dionese et al. (43)	2023	NR	Retrospective	72	adjuvant therapy	OS	1375	UC
Xinping Yi et al. (32)	2023	NR	Retrospective	496	surgery+adjuvant therapy	RFS	525	NMIBC

OS^a^, overall survival; CSS^b^, cancer-specific survival; RFS^c^, recurrence-free survival; UCB^d^, urothelial carcinoma of the bladder; UTUC^e^, upper tract urothelial cancer; NMIBC^f^, non-muscle-invasive bladder cancer; MIBC^g^, muscle-invasive bladder cancer; NR, Not reported.

### 3.2 Quality assessment

The quality assessment of the cohort studies was conducted using the modified Newcastle-Ottawa Scale (NOS), resulting in scores that ranged between 6 and 8, indicating a robust methodological quality across the studies (**Table 2**).

**Table 2:** Quality score of included studies based on the NOS scale.

Study	Selection			Comparability			Exposure			Total stars
	^a^REC	^b^SNEC	^c^AE	^d^DO	^e^SC	^f^AF	^g^AO	^h^FU	^i^AFU	
Grossmann NC et al. (23)	1	1	1	1	1	1	1	1		8
Kobayashi S et al. (40)	1	1	1	1	1		1	1		7
Mori K et al. (28)	1	1	1	1	1		1	1	1	8
Chien TM et al. (39)	1	1	1	1	1		1	1	1	8
Katayama S et al. (25)	1	1	1	1	1	1	1	1		8
Zhang et al. (50)	1	1	1	1	1	1	1			7
Huang et al. (51)	1	1	1	1	1	1	1	1		8
Jan HC et al. (24)	1	1	1	1	1		1			6
Zheng Y et al. (37)	1	1	1	1	1		1	1	1	8
Xingxing Tang et al. (30)	1	1	1	1	1		1	1		7
Wentao Zhang et al. (49)	1	1	1	1	1		1	1	1	8
Zhi-Bin Ke et al. (26)	1	1	1	1	1		1	1		7
Peng Liu et al. (27)	1	1	1	1	1		1	1		7
Huifeng Bi et al. (38)	1	1	1	1	1		1	1	1	8
Li Deng-Xiong et al. (21)	1	1	1	1	1		1	1		7
G Fornarini et al. (44)	1	1	1	1	1	1	1	1		8
Ali Yılmaz et al. (42)	1	1	1	1	1	1	1	1		8
Shimpei Yamashita et al. (41)	1	1	1	1	1	1	1	1		8
Shiyu Zhang et al. (34)	1	1	1	1	1	1	1	1		8
Li Ding et al. (22)	1	1	1	1	1		1	1		7
Patrik Palacka et al. (47)	1	1	1	1	1		1	1	1	8
Sacit Nuri Gorgel et al. (45)	1	1	1	1	1		1	1	1	8
Chengbo Wang et al. (31)	1	1	1	1	1		1	1		7
Hasan Yilmaz et al. (33)	1	1	1	1	1		1	1	1	8
Ruining Zhao et al. (36)	1	1	1	1	1		1	1	1	8
Abolfazl Salari et al. (48)	1	1	1	1	1		1	1	1	8
Zhenkai Luo et al. (46)	1	1	1	1	1		1	1		7
Pierluigi Russo et al. (29)	1	1	1	1	1		1	1		7
Xiaoping Zhang et al. (35)	1	1	1	1	1		1	1	1	8
Michele Dionese et al. (43)	1	1	1	1	1	1	1	1		8
Xinping Yi et al. (32)	1	1	1	1	1		1	1	1	8

a REC, representativeness of the cohort;

b SNEC, selection of the none posed cohort;

c AE, ascertainment of exposure;

d DO, demonstration that outcome of interest was not present at start of study;

e SC, study controls most important factors;

f AF, study controls for other important factors;

g AO, assessment of outcome;

h FU, follow-up long enough for outcomes to occur;

i AFU, adequacy of follow-up of cohort (≥ 80%).

### 3.3 Recurrence-free survival

**Figure 2:**
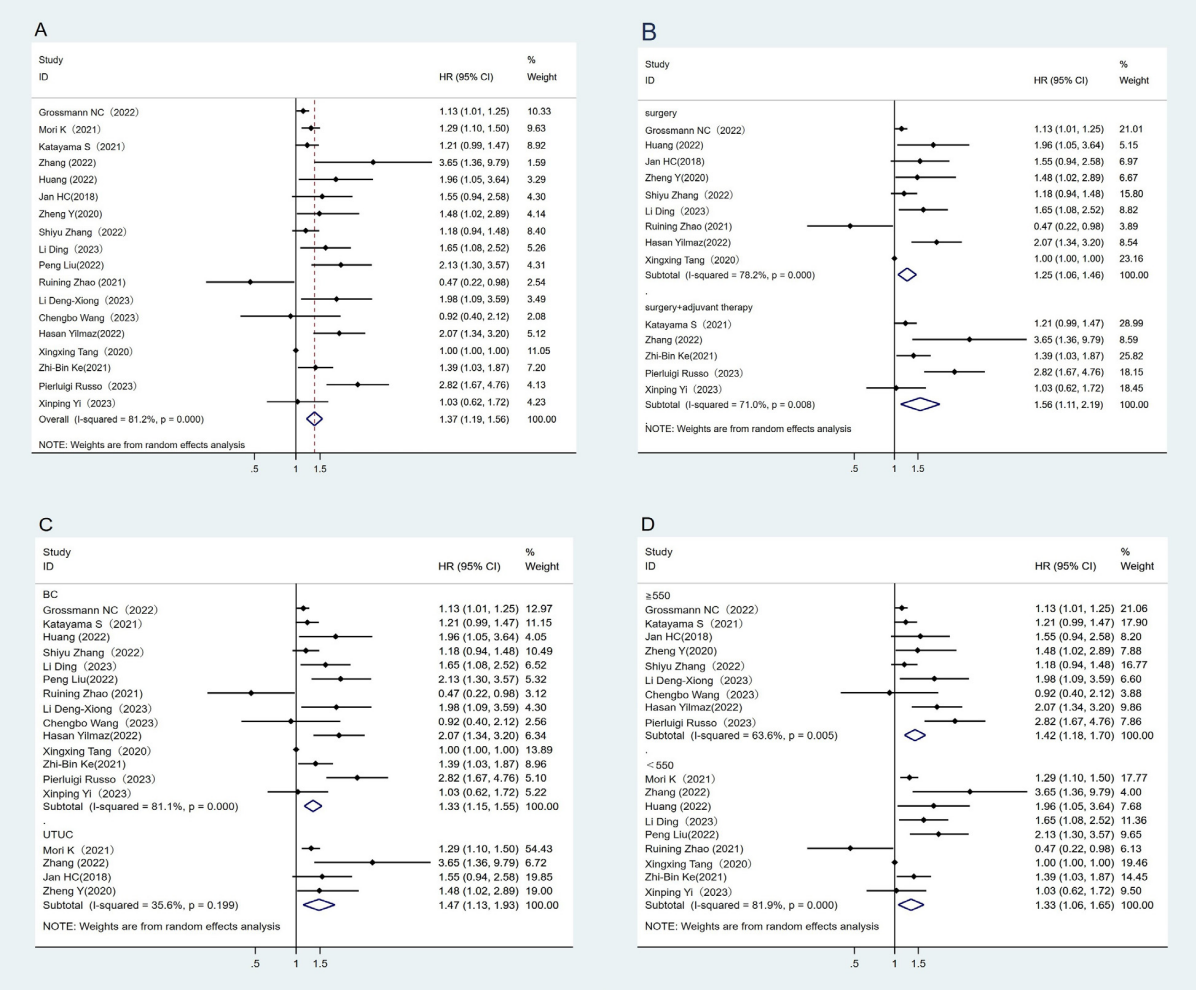
Forest plot and meta-analysis of the RFS between low and high SII. **(A)** Overall. **(B)** According to the treatment methods. **(C)** According to the tumor location. **(D)** According to the SII cutoff value.

A total of 18 studies have reported the association between RFS and SII (21–37, 50, 51). The heterogeneity test showed high heterogeneity among studies (I^2^ = 81.2%, P < 0.05). The results of the meta-analysis showed that RFS was better in the low SII group than in the high SII group, indicating that patients with high SII had shorter RFS (HR = 1.37, 95%CI (1.19, 1.56), P < 0.05) (**Figure 2A**). At the same time, subgroup analysis conducted in this study showed that higher SII was associated with poorer RFS (p<0.05) regardless of treatment regimen, tumor type, or SII cut-off, and high SII was an important prognostic biomarker for poorer RFS in the UC population (**Figures 2B**–**D**).

### 3.4 Cancer-specific survival

**Figure 3:**
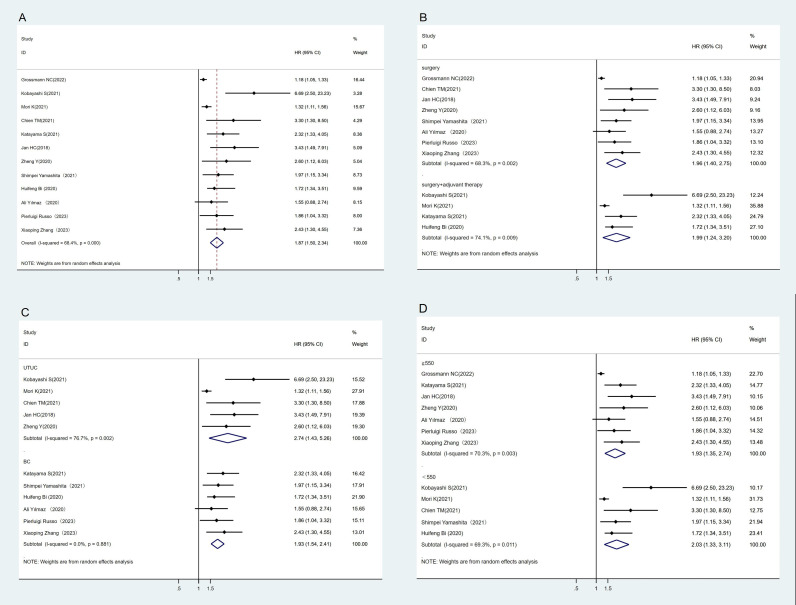
Forest plot and meta-analysis of the CSS between low and high SII. **(A)** Overall. **(B)** According to the treatment methods. **(C)** According to the tumor location. **(D)** According to the SII cutoff value.

A total of 12 studies have reported an association between CSS and SII (23–25, 28, 29, 35, 37–42). The heterogeneity test showed high heterogeneity among studies (I^2^ = 68.4%, P < 0.1). The results of meta-analysis showed that the lower SII group had better CSS than the higher SII group (HR = 1.87, 95%CI (1.50, 2.34), P < 0.05) (**Figure 3A**). We also performed subgroup analyses where higher SII was associated with poorer CSS, regardless of treatment regimen, tumor type, or SII cut-off (**Figures 3B**–**D**).

### 3.5 Overall survival

**Figure 4:**
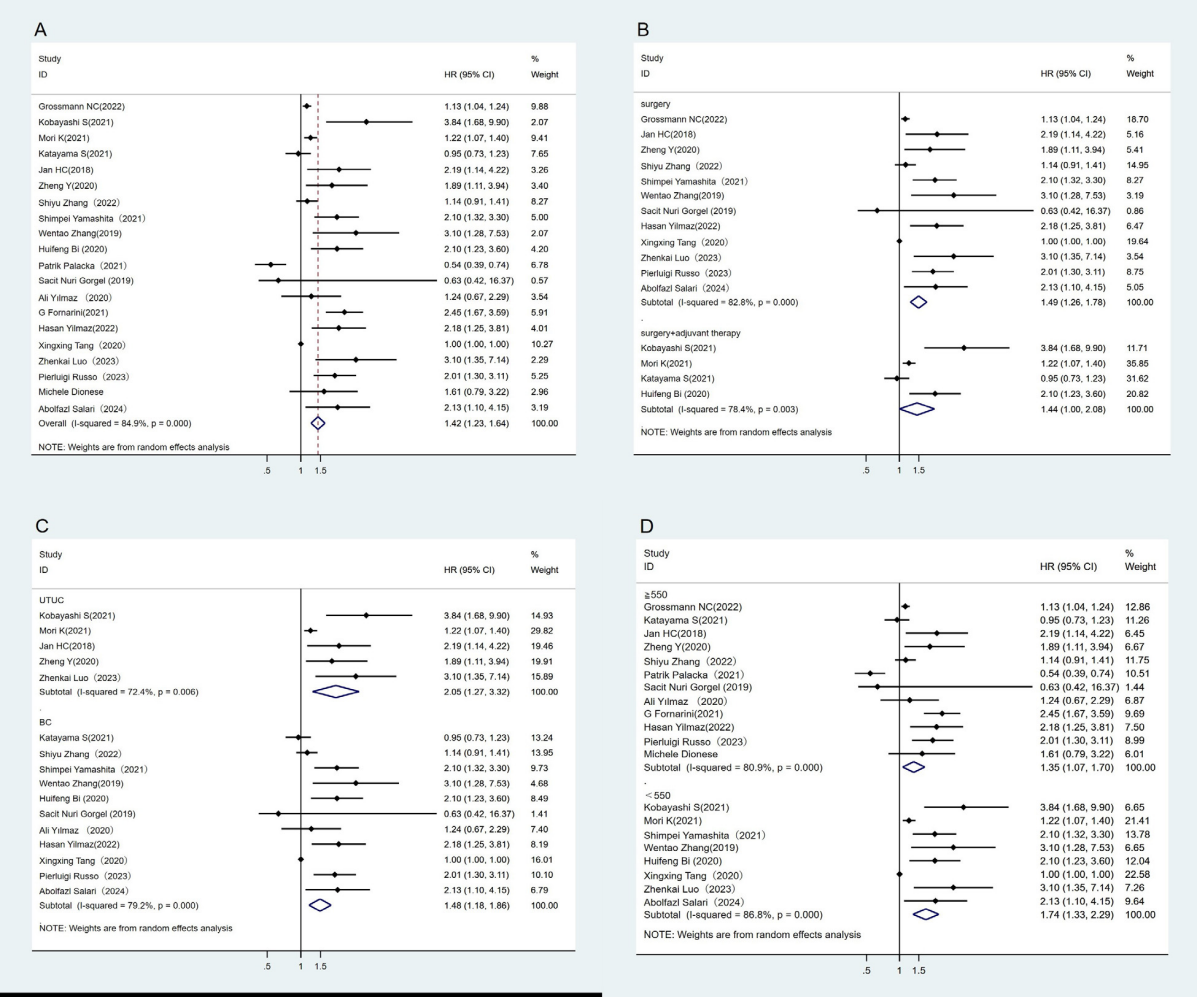
Forest plot and meta-analysis of the OS between low and high SII. **(A)** Overall. **(B)** According to the treatment methods. **(C)** According to the tumor location. **(D)** According to the SII cutoff value.

A total of 20 studies have reported the association between OS and SII (23–25, 28–30, 33, 34, 37, 38, 40–49). The heterogeneity test showed high heterogeneity between studies (I^2^ = 84.9%, P < 0.1). The results of meta-analysis showed that the OS in the low SII group was better than that in the high SII group. (HR = 1.42, 95%CI (1.23, 1.64), P < 0.05) (**Figure 4A**). We also performed subgroup analyses where higher SII was associated with poorer OS, regardless of treatment regimen, tumor type, or SII cut-off (**Figures 4B**–**D**).

## 4 Sensitivity analysis and publication bias

Despite the inclusion of high-quality studies following stringent quality assessment, there was an inevitably high degree of heterogeneity between studies. We used sensitivity analysis to track the sources of heterogeneity for each outcome measure. The test showed no significant changes in the overall HR estimates for these survival outcomes, suggesting that the findings of the meta-analyses were robust and stable (**Supplementary Figures S1**–**S3**).

Begg’s test and funnel plots were used to assess publication bias in the included studies. Visual examination of the funnel plot revealed asymmetry, indicating a higher possibility of publication bias (**Supplementary Figures S4**–**S6**).

## 5 Discussion

Inflammation plays an important role in the biological behavior of tumors. Inflammatory cells in the tumor microenvironment participate in various proinflammatory responses. The number of immune cells and other components in the tumor microenvironment play an important role in the occurrence, malignant transformation, development, and metastasis of tumors (52). Immune cells in the tumor microenvironment, such as T cells, macrophages, and dendritic cells, as well as inflammatory cells, such as neutrophils and lymphocytes, are involved in tumor development and immune responses. The tumor microenvironment directly or indirectly affects tumor cell proliferation, migration, and angiogenesis by releasing various inflammatory mediators (53, 54). Long-term exposure to inflammatory cytokines can promote cell proliferation and angiogenesis (55), whereas DNA damage and excessive production of reactive oxygen species (ROS) stimulate tumor growth (56). Previous meta-analyses found that a high SII is independently associated with poor oncological outcomes in patients with renal cell carcinoma and colorectal cancer (57, 58). High SII values are associated with poor outcomes in patients with rectal cancer, including reduced OS and disease-free survival (59). An elevated SII is associated with poor OS in many solid tumors. The SII can act as a powerful prognostic indicator of poor outcomes in patients with solid tumors (60).

Research has established that chronic inflammation is widely involved in tumor occurrence and progression. Tumor-associated systemic inflammatory responses involve inflammatory cells and various inflammatory mediators (61). Our study revealed that a low SII was associated with better OS, RFS, and CSS, which is similar to the findings of previous studies. It has been suggested that this advantage can be explained by the function of neutrophils and lymphocytes and has been shown to be associated with oncological outcomes in several types of cancers (60, 62). The SII contains three types of peripheral blood inflammatory biomarkers based on platelet count (P), neutrophils (N), and lymphocytes (L) using the following formula: SII = P × N/L (63). SII can reflect the balance of inflammation and immune response better than a single marker (64) The combination of three blood components gives a more complete picture of the body’s immune and inflammatory state. For example, traditional inflammatory markers such as ESR (erythrocyte sedimentation rate) and CRP (C-reactive protein) may be interfered with by various factors such as malignant tumors and drugs, and have certain limitations. All data used for the calculation can be obtained from routine blood tests, which means that researchers can collect and analyze the SII data (58). It is a noninvasive measurement with the advantages of simplicity, ease of detection, low cost, and ease of analysis, and is suitable for promotion and use in primary medical institutions. Moreover, the calculation formula of SII is simple, easy to be quickly calculated and applied in clinical practice, does not increase the burden on patients, does not require additional laboratory tests or expensive reagents, and is highly cost-effective. Compared with some emerging serological assessment tools, such as liquid biopsy, although it has higher sensitivity and specificity, it has higher requirements for testing equipment and testing technology, and it is difficult to widely promote and apply in medical institutions in the short term (65).

SII can fluctuate based on a patient’s condition, tumor burden, and immunoinflammatory response status, thereby aiding in the monitoring of disease progression and treatment response. Research has demonstrated that SII serves as an independent prognostic factor across various tumors (including liver cancer, stomach cancer, colorectal cancer, etc.) (59, 66, 67), Its predictive capability surpasses that of conventional parameters such as the neutrophil to lymphocyte ratio (NLR) and the platelet to lymphocyte ratio (PLR). Additionally, SII is less influenced by a patient’s hydration status, rendering it more reliable for assessing the immunoinflammatory state of patients. Moreover, SII exhibits greater stability and reliability in diverse clinical scenarios due to its reduced sensitivity to fluid load compared to other indicators. Currently, SII is extensively utilized for predicting patient prognosis across multiple cancers-encompassing overall survival and disease-free survival—thus providing valuable prognostic evaluation metrics. Compared with some traditional tumor markers such as AFP (alpha-fetoprotein), which plays an important role in predicting prognosis, some tumor patients still show negative AFP, and the increase of AFP may also be related to other non-neoplastic diseases (68). Therefore, AFP alone has limitations in evaluating the prognosis of tumor patients.

Although the threshold at which SII predicts prognosis varies from study to study, the results show that the higher the SII, the worse the prognosis, which provides an important reference for clinical decision-making. A high SII score indicates an enhanced inflammatory response or a weakened immune response. An increase in the SII indicates an increase in the number of neutrophils and platelets, which leads to enhanced tumor cell growth, reproduction, and metastasis. Concurrently, a reduction in lymphocyte count results in a diminished capacity of the immune system to combat tumors. Lymphocytes, particularly T lymphocytes, are important weapons in antitumor immune responses (69). Lymphocytopenia is usually accompanied by leukocytosis and thrombocytosis, which may help tumor cells evade immune surveillance and prevent damage to the autoimmunity of cytotoxic T cells. A high SII reflects changes in the cancer microenvironment that are conducive to cancer occurrence, progression, and metastasis (70). SII is closely related to the prognosis of various tumors (such as hepatocellular carcinoma, colorectal cancer, and renal cell carcinoma) and can be used as an independent prognostic factor. Because of its simplicity, economy, noninvasiveness, and potential predictive value, the SII shows broad prospects for clinical applications. A high SII may indicate a strong inflammatory response and immune suppression, which are related to immune escape and tumor progression. Tumor cells alter the tumor microenvironment by secreting cytokines and chemokines to promote their growth, invasion, and metastasis.

To find out the accurate effect of SII on the prognosis of urothelial carcinoma, we conducted a meta-analysis including 31 articles and 14,437 patients to investigate the association between SII status and the prognosis of urothelial carcinoma. A high SII was an independent predictor of RFS, CSS, and OS in patients with urothelial carcinoma.

A high SII was associated with poor OS, RFS, and CSS in patients with urothelial carcinoma, and the clinical features indicated that the cancer was more malignant. This is in line with the results of another study: compared with the detailed subgroup analysis in this paper, patients with a low SII had better OS in UTUC based on the tumor location (12). SII may help predict how patients with cancer respond to treatments, including surgery, chemotherapy, radiation, and immunotherapy. In some cases, a high SII is associated with adverse reactions to certain treatments. These results suggest that the SII can play an important role as an effective factor for poor prognosis and guide the clinical treatment of patients with urothelial carcinoma. However, due to the limitations of this study, further high-quality studies are required to verify our results.

Our meta-analysis has several limitations. First, an optimal SII threshold was not determined. The included studies used different critical thresholds, which may have led to heterogeneity among the studies. There is no standard value; therefore, the conclusions may differ. Second, the included studies were retrospective rather than prospective. The original data inevitably have limitations and deviations that reduce the strength of the argument, which may lead to selection bias. Further prospective studies are required to confirm this. Third, our meta-analysis included only qualified published studies in English or Chinese and did not include relevant articles in other languages, which may also lead to inherent heterogeneity. Fourth, the SII cutoff values were inconsistent, which may have led to heterogeneity. Fifth, most of the studies were conducted in Asia, and the results may be more relevant to Asian patients. The sample sizes also varied significantly. The relatively small sample size led to the relatively low reliability of this study. The clinical application of the SII in urothelial carcinoma has shown a close relationship with tumor prognosis. Future studies should explore the role of the SII in different tumor types and validate its application in individualized treatment strategies.

## 6 Conclusion

This meta-analysis showed that elevated SII before treatment was associated with OS, RFS, and CSS in patients with urothelial carcinoma. Therefore, SII monitoring may be an effective method for improving the survival rate of patients with urothelial carcinoma. Well-designed, large-scale prospective studies should be conducted to evaluate and verify the correlation between SII and prognosis in patients with urothelial carcinoma.

## Data Availability

The original contributions presented in the study are included in the article/**Supplementary Material**. Further inquiries can be directed to the corresponding author/s.

## Supplementary material

Supplementary Figure 1 Sensitivity analysis of the RFS between low and high SII.

Supplementary Figure 2 Sensitivity analysis of the CSS between low and high SII.

Supplementary Figure 3 Sensitivity analysis of the OS between low and high SII.

Supplementary Figure 4 Publication bias funnel plot for RFS.

Supplementary Figure 5 Publication bias funnel plot for CSS.

Supplementary Figure 6 Publication bias funnel plot for OS.
