# Supplementary figures and images for: Prognostic significance of systemic immune inflammation index in patients with urothelial carcinoma: a systematic review and meta-analysis
Source: Front Oncol. 2024 Dec 23;14:1469444. doi: 10.3389/fonc.2024.1469444 (PMC11700819; doi:10.3389/fonc.2024.1469444)

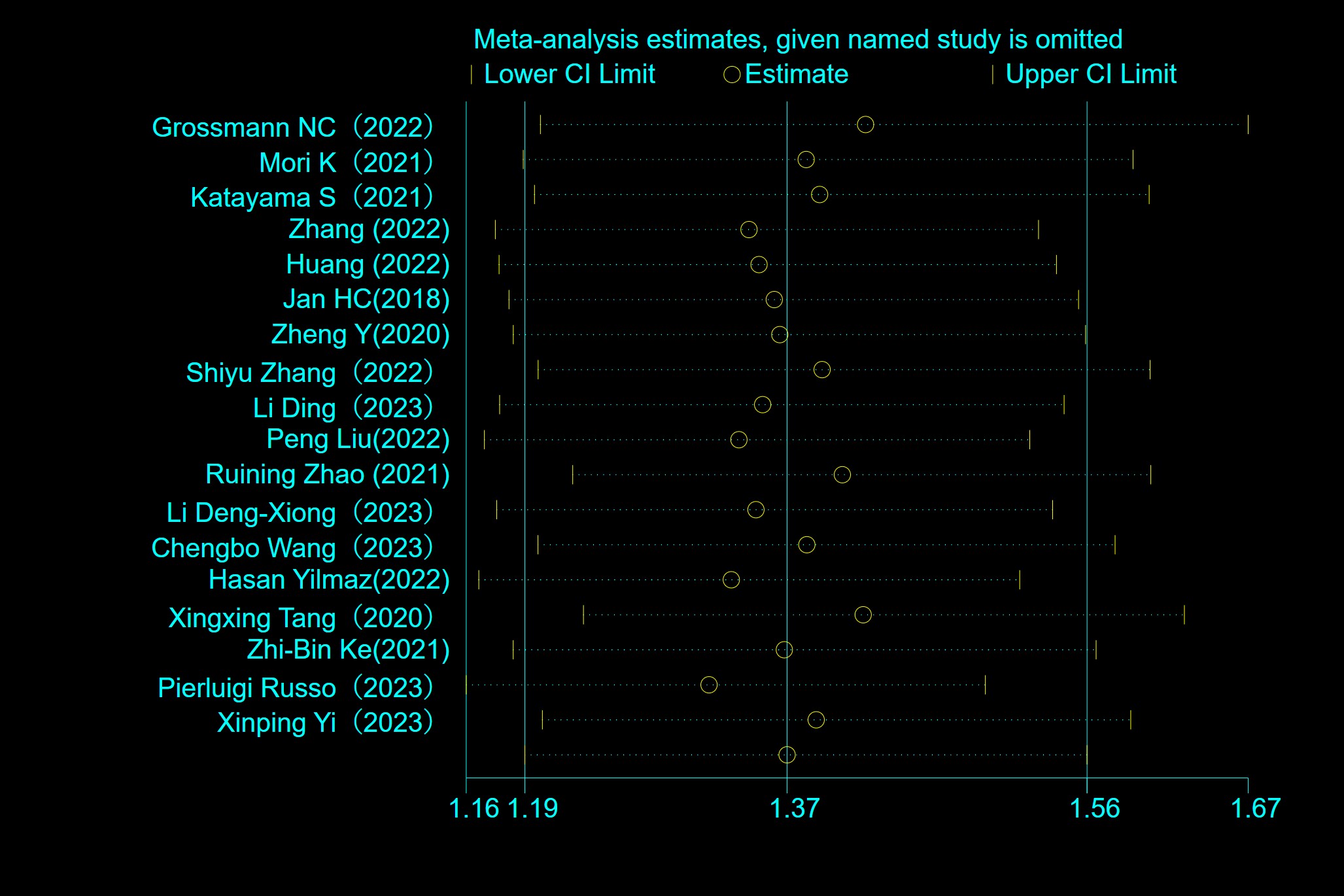

Supplement: Supplementary Figure 1 — Sensitivity analysis of the RFS between low and high SII. [file Image1.jpeg]

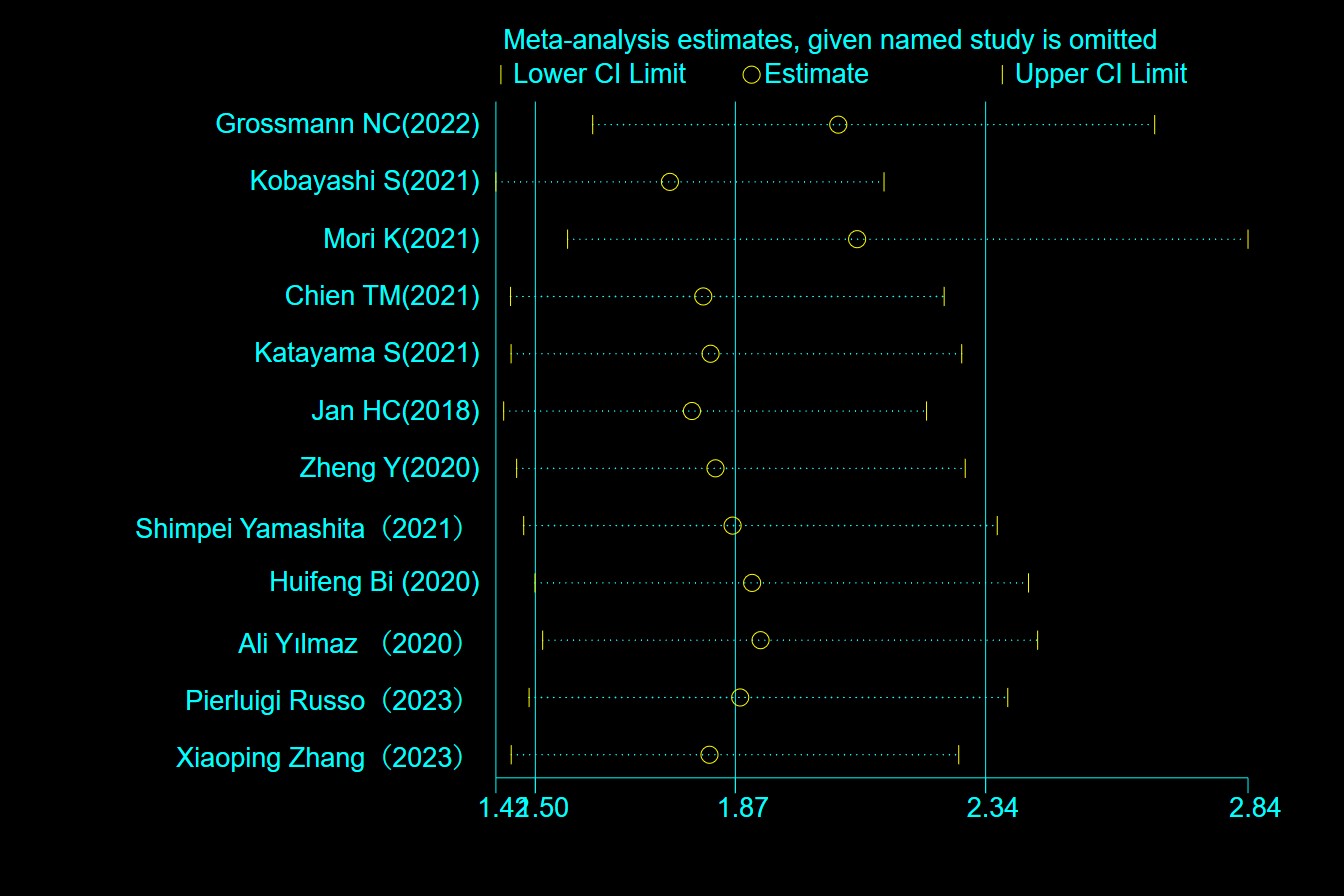

Supplement: Supplementary Figure 2 — Sensitivity analysis of the CSS between low and high SII. [file Image2.jpeg]

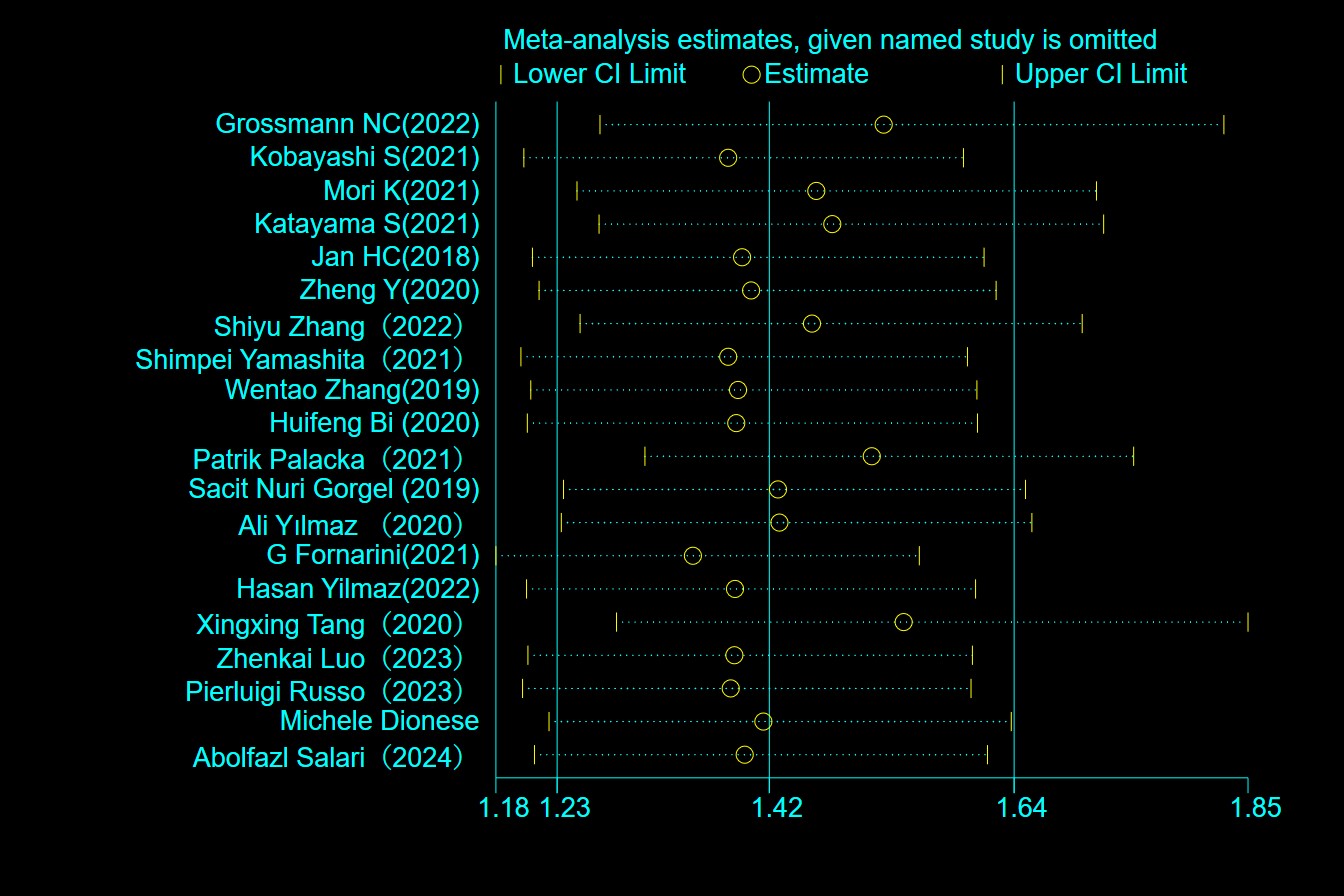

Supplement: Supplementary Figure 3 — Sensitivity analysis of the OS between low and high SII. [file Image3.jpeg]

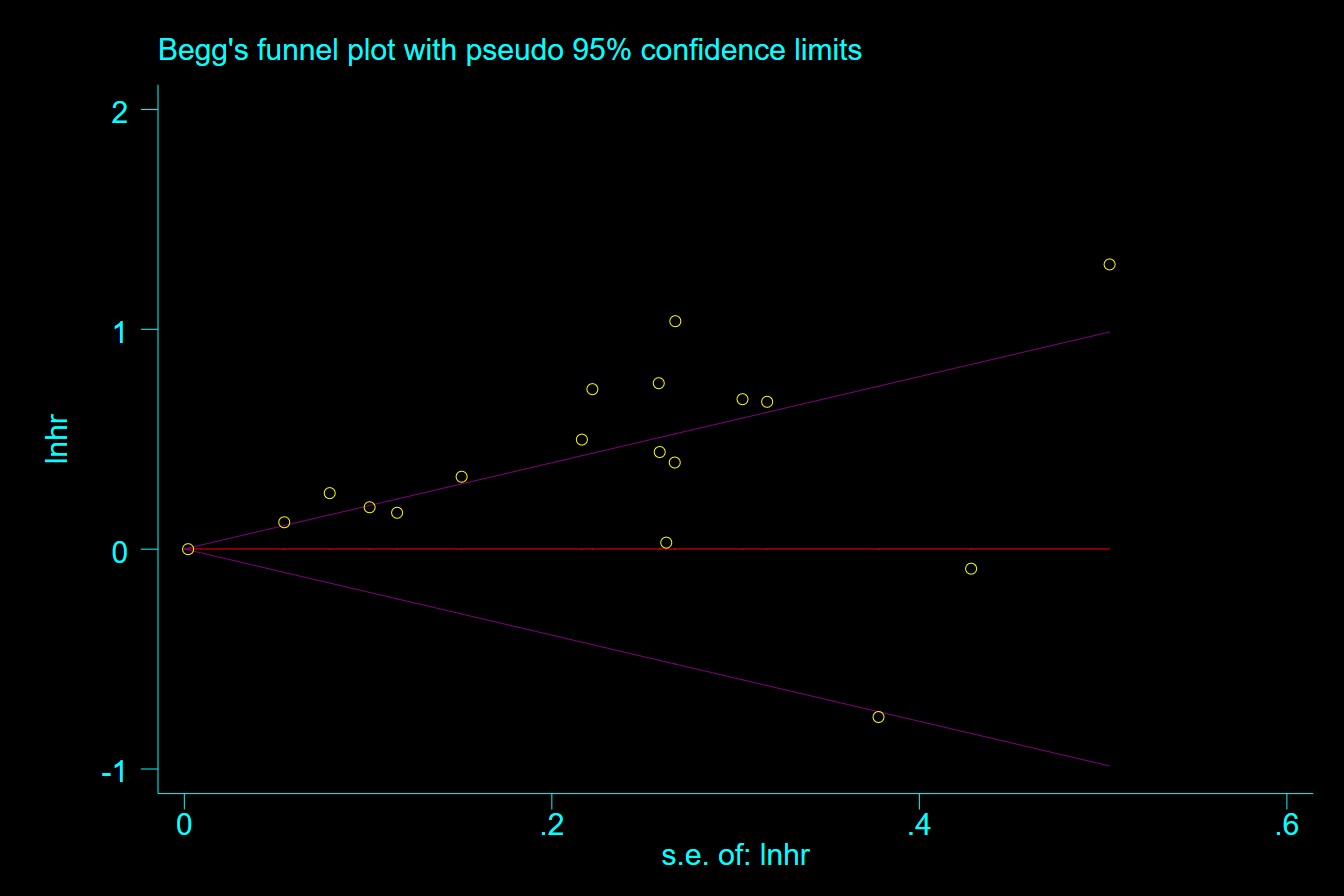

Supplement: Supplementary Figure 4 — Publication bias funnel plot for RFS. [file Image4.jpeg]

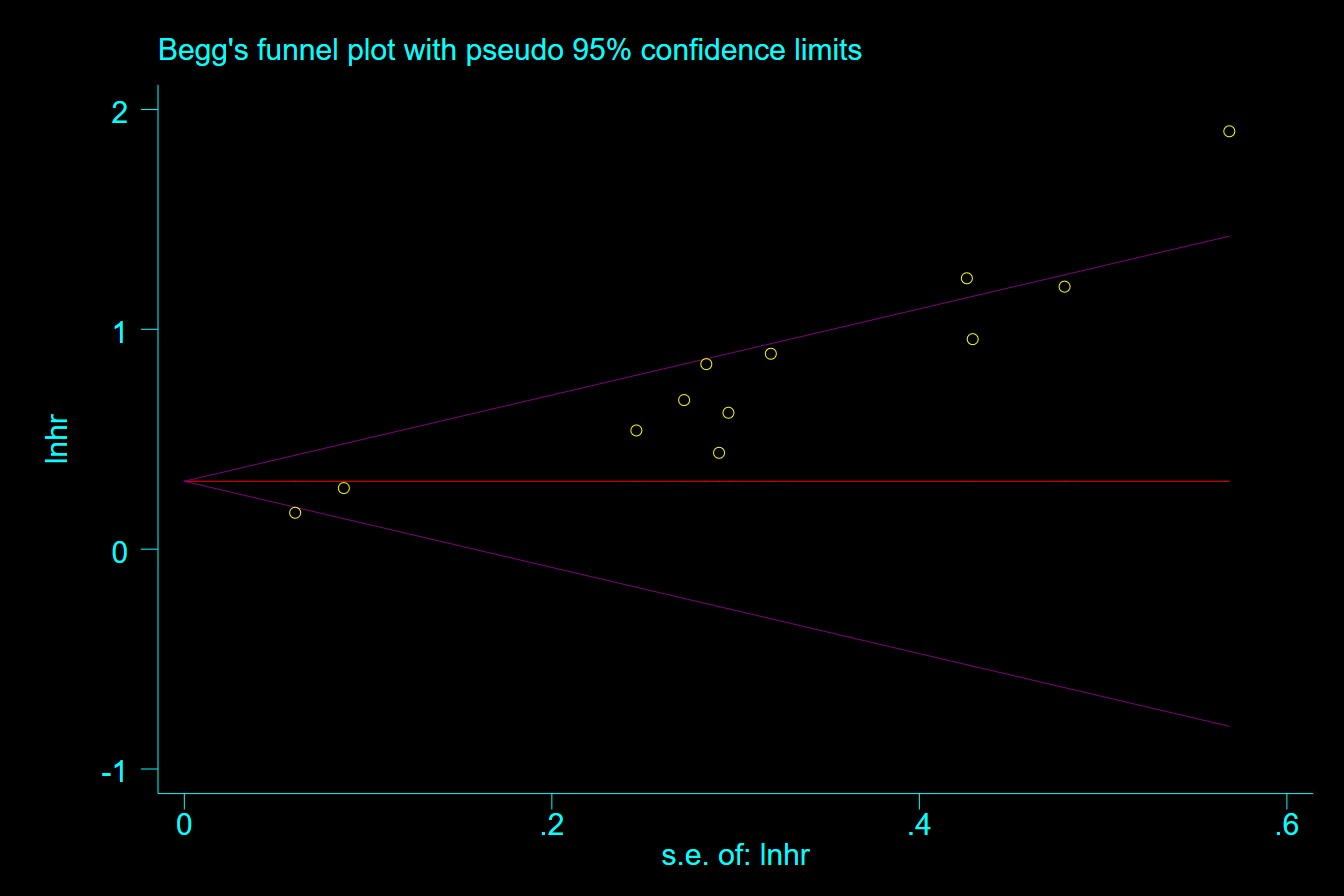

Supplement: Supplementary Figure 5 — Publication bias funnel plot for CSS. [file Image5.jpeg]

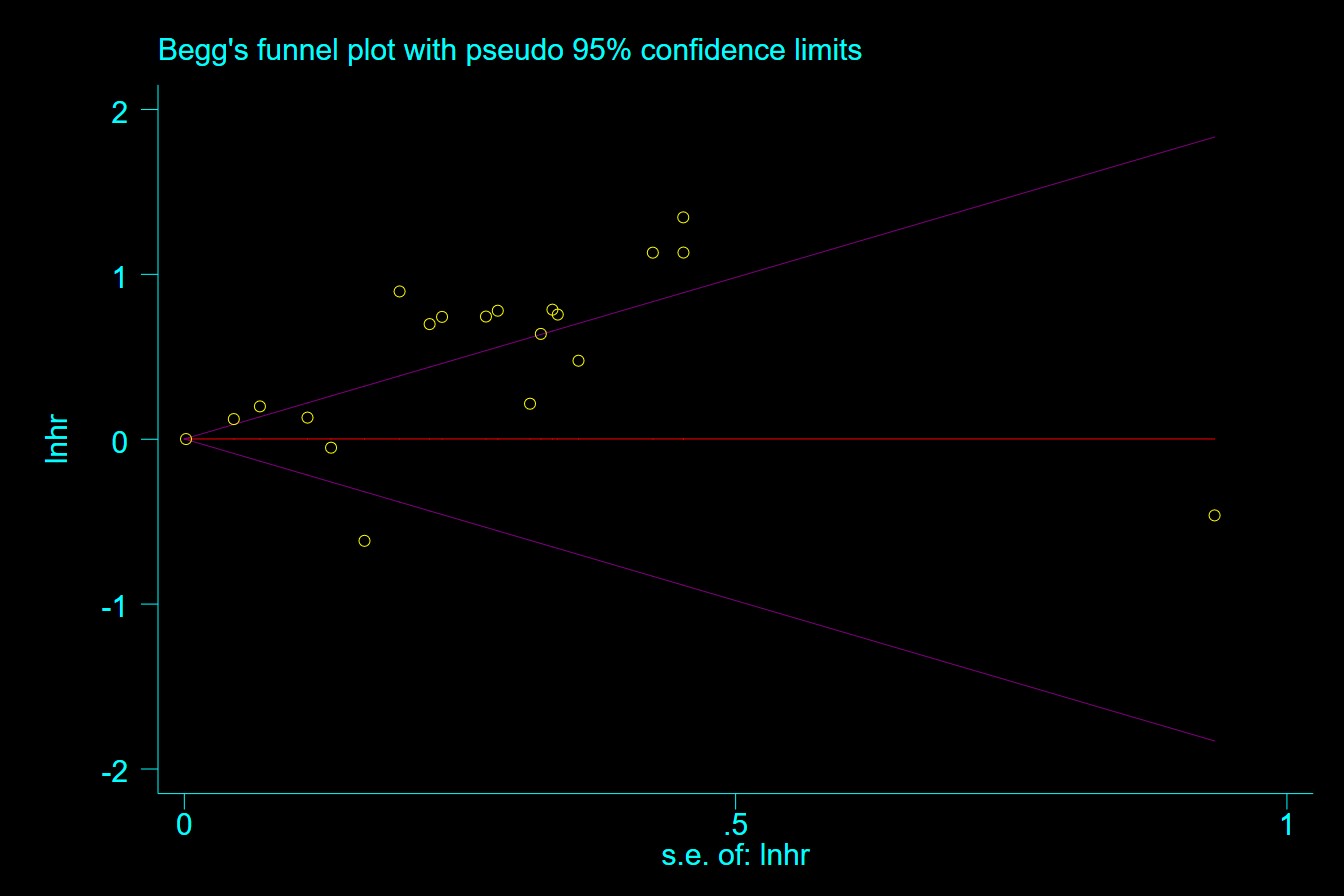

Supplement: Supplementary Figure 6 — Publication bias funnel plot for OS. [file Image6.jpeg]
